# A Comparison of Quantitative Pupillometry and VOMS in Division 1 Female Soccer Players

**DOI:** 10.3390/medicina61061109

**Published:** 2025-06-19

**Authors:** John Duane Heick

**Affiliations:** Department of Physical Therapy and Athletic Training, Northern Arizona University, Flagstaff, AZ 86011, USA; john.heick@nau.edu

**Keywords:** concussion baseline testing, ocular motor function, pupillary light reflex, pupillometry

## Abstract

*Background and Objectives:* Vision uses about half of the pathways within the brain, and these anatomical structures are susceptible to injury in concussion. Authors have suggested that subconcussive head impacts, common in soccer, may disrupt visual function. The following study aimed to explore and compare quantitative pupillometry and Vestibular Ocular Motor Screening (VOMS) in female soccer athletes. *Materials and Methods:* Twenty-six Division 1 female soccer athletes (20.46 ± 2.36 years) received baseline quantitative pupillometry and VOMS measurements. *Results:* Of the 26 tested athletes, 3 (11.5%) had clinically significant pupillometry findings at baseline. The mean Neurological Pupil Index or NPi, a composite generated from pupillometry, did not vary: 3.9 ± 0.4 (right eye) and 4.0 ± 0.4 (left eye). No difference in NPi was observed compared to the VOMS score (*p* > 0.05). Kruskal–Wallis H tests were significant in the right eye for constriction percentage (*χ*^2^(2) = 17.843, *p* < 0.001, *E*^2^ = 0.69) and minimum pupil size (*χ*^2^(2) = 7.976, *p* = 0.019, *E*^2^ = 0.31). A post hoc Dunn test showed significant differences in constriction percentage and minimum pupil size between low NPi and high NPi groups (*p* < 0.05). One athlete sustained a concussion. NPi was measured within 24 h and was normal, but VOMS was not (total score = 4). *Conclusions:* The components of pupillometry need more investigation, and there is a need for agreement on concussion-specific cutoffs for quantitative pupillometry for concussion assessment. The lack of a relationship between quantitative pupillometry and VOMS suggests that these tools evaluate different constructs. Athletes with an NPi < 3.8 had significantly less constriction percentage and larger minimum pupil size than athletes with higher NPi scores. More research should be carried out to determine the usefulness of the NPi score, and perhaps researchers should consider individual pupillometry components.

## 1. Introduction

Soccer athletes experience an increased risk of concussion due to repetitive forces when heading the ball [1]. These subconcussive blows may lead to neuropathological changes in the brain [2]. In professional soccer players, the number of concussions and headers was inversely related to neurocognitive test scores for visual and verbal memory. Due to methodological limitations, evidence remains inconclusive on the effect of subconcussive blows [3]. The common mechanisms of concussion injuries include player-to-player, player-to-ground, and player-to-object contact, such as the goal post or the ball. Increased rates of concussions have been observed in female athletes compared to their male counterparts in the same sport [4].

National Collegiate Athletic Association (NCAA) female soccer athletes experience the highest incidence of concussions at 0.63 concussions per 1000 athletic exposures, according to the NCAA Injury Surveillance System [5]. Female NCAA soccer athletes were at an almost 2.5 times higher risk for concussions after impact with either the ball or goal post compared to males [6]. In one group of Division 1 female soccer players, headers accounted for 20% of concussions [7]. Female soccer athletes may be at a higher risk of injury when performing headers due to having lower anthropomorphic measures and decreased neck size and strength [1,4]. In comparing male and female collegiate soccer athletes, females had 15% less head–neck segment mass and approximately 50% less isometric neck flexor and extensor strength [8]. In Division 1 soccer athletes, women had decreased neck girth and neck flexion strength than males [9]. Female soccer players have displayed higher peak linear and rotational acceleration than males during purposeful heading, possibly putting them at risk for increased shearing forces [10]. Decreased anthropomorphic measures and neck strength and girth were correlated with increased head acceleration in soccer players [8,10].

Female athletes may also be at risk for increased symptoms and prolonged recovery after concussion [11]. Collegiate female soccer athletes have shown prolonged neuropsychological impairments up to 8 months after sustaining a concussion [12]. Female athletes and those with a personal or family history of motion sickness have shown increased vestibular and ocular symptoms post-concussion, as assessed via Vestibular Ocular Motor Screening (VOMS) [13]. VOMS is a reliable tool for screening concussions in college athletes [14] and has adequate diagnostic power (sensitivity = 0.96; specificity = 0.46). Scoring is based on subjective symptoms (e.g., headache, dizziness, nausea, fogginess) rated on a 10-point scale as reported by the patient after completing each vision/vestibular test. A score of ≥2 on any test would be considered clinically significant [15]. The near point of convergence (NPC) is the only objective component of the VOMS, in which the distance from the bridge of the nose to the point where the patient sees a double object or exhibits exotropia is measured. However, NPC measurements can vary depending on the target that is used [16,17]. Additionally, there is disagreement about the clinical cutoff, with different disciplines suggesting < 5 cm, <6 cm, or <10 cm [15,16,18,19]. Due to the interaction of systems, a multifaceted approach has been suggested for concussion assessment.

Biomarkers for concussion provide objective diagnostic criteria [20]. Authors suggest quantitative pupillometry as a potential biomarker for concussion [1,2,21]. Quantitative pupillometry consists of a range of objective measures that vary by the pupillometer that is used. Standard components include pupil size, latency, constriction percentage, constriction velocity, and dilation velocity. Additionally, some devices produce a Neurological Pupil index (NPi) score, which provides an index score based on the quantitative pupillometry components compared to normative values that range from 0 to 5 [22]. A clinical cutoff score of <3.0 or a difference between the eyes of >0.7 for NPi has been suggested [22]. Researchers have suggested a cutoff of <0.8 mm/s for constriction velocity [23] and <0.86 mm/s for dilation velocity [20]. Significant differences have been identified within pupillometry components after injury, with constriction percentage, constriction velocity, and dilation velocity showing the most consistent differences when comparing controls to concussed individuals [20,21,22,24,25].

Researchers have observed abnormal quantitative pupillometry results in adolescents with a concussion on all measures except for latency compared to healthy controls. Changes in constriction percentage, maximum constriction velocity, and dilation velocity compared to baseline were seen in adolescent football players after asymptomatic high-acceleration head impacts. Significant changes were observed in constriction and maximum constriction velocity throughout the season [21]. Adults in neurological and critical care units with severe traumatic brain injuries who had an abnormal NPi score were more likely to have increased ICP [22]. Military personnel with blast-induced or other forms of mild traumatic brain injury have shown decreased constriction and dilation velocity compared to healthy age-matched controls [20]. There needs to be more research about quantitative pupillometry in collegiate athletic populations. This study aimed to explore and compare quantitative pupillometry and VOMS in Division I female soccer athletes. A secondary aim was to compare quantitative pupillometry and VOMS pre- and post-concussion.

## 2. Materials and Methods

### 2.1. Study Design

#### 2.1.1. Participants

Division 1 female soccer athletes aged 18 to 28 were recruited during concussion baseline testing. Athletes were excluded if they had a lower extremity injury in the past three months that caused them to miss more than one day of practice; a head injury in the past six months; or were diagnosed with a visual, vestibular, or balance disorder. The rationale for these exclusion criteria was that if an athlete had a concussion, the athlete would already have abnormal results.

#### 2.1.2. Procedures

Athletes who agreed to participate completed the quantitative pupillometry and the VOMS performed by the author. Athletes were tested at baseline and after sustaining a concussion. Testing was completed by the investigators for baseline testing and for athletes who sustained a concussion during the season. All testing was performed in the soccer-specific training room under the same conditions, including the amount of light and the time of day.

##### Vestibular Ocular Motor Screening (VOMS)

VOMS is a validated tool for concussion assessment consisting of five vision and vestibular tests: smooth pursuits, horizontal and vertical saccades, near point of convergence (NPC), horizontal and vertical vestibular-ocular reflex, and visual motion sensitivity [15]. Smooth pursuit is the ability to track a slow-moving target with the eyes while the head remains stationary. The saccade movement is the ability of the eyes to move quickly between targets. Convergence is the ability to view a close target without double vision. The vestibular-ocular reflex is the ability to stabilize vision while the head moves. Visual motion sensitivity tests the ability to inhibit vestibular-induced eye movements. The procedures for this assessment were standardized per Mucha et al. [15]. Athletes were seated for all tests, except for the visual motion sensitivity test, which requires participants to stand. Athletes rated their symptoms on a scale from 0 to 10 at baseline and after each test.

#### 2.1.3. Quantitative Pupillometry

The NeurOptic^®^ NPi-200 pupilometer (NeurOptics, Irvine, CA, USA) was used to assess quantitative pupillometry. Eight output measures are produced: (1) NPi (composite score determined by an algorithm), (2) pupil size (resting pupil size), (3) minimum pupil size (pupil size during peak constriction), (4) constriction percentage (pupil size divided by minimum pupil size), (5) constriction velocity (amount of constriction divided by time resulting in average velocity), (6) maximum constriction velocity (peak velocity during constriction), (7) latency (time difference between light stimulation and the onset of pupillary constriction), and (8) dilation velocity (pupil recovery after constriction divided by the recovery time). The NPi is a graded score that creates standardized z-scores for each pupillometry component and produces an NPi score of 0 to 5. An NPi score of less than three is considered abnormal, or a difference between 0 and 0.7 between both eyes is considered clinically significant [26]. Participants were seated during standardized testing and asked to focus on a target 10 feet away. The NeurOptic^®^ NPi-200 pupilometer was placed over one eye, flashing light at a set distance from the pupil to trigger pupil constriction and to allow the measurement of pupil responses. The test was performed twice in the left and right eye, with a break of 30 s between each test. Each athlete took approximately 3 min to complete the test. The NPi scores were categorized into low, medium, or high percentiles.

### 2.2. Statistical Analysis

Descriptive statistics were calculated for VOMS and quantitative pupillometry. Pearson correlations were used to determine which quantitative pupillometry components had the strongest correlation with the NPi score. Percentile cutoffs were used to determine low, medium, and high scores for NPi, similarly to Oddo et al. [26]. Normality was assessed using the Shapiro–Wilk test. Nonparametric Kruskal–Wallis H tests were performed to determine between-group differences for NPi, NPC, and VOMS scores. Post hoc Dunn tests with a Bonferroni correction were utilized to determine where differences existed between pairwise comparisons when significant. Variance and effect sizes were calculated for significant Kruskal–Wallis H tests using eta-squared (η^2^) and epsilon squared (*E*^2^), respectively. A significance level of *p* < 0.05 was used for all analyses. This study was approved by the Northern Arizona University Institutional Review Board (#1439398-1).

## 3. Results

Twenty-six Division 1 female soccer athletes (20.46 ± 2.36 years) were screened at baseline for quantitative pupillometry and VOMS (Table 1). Three athletes (11.5%) had clinically significant quantitative pupillometry findings (NPi < 3.00 or 0.7 difference between eyes). Two athletes had an NPi score of 3.00, and one had an NPi score difference of >0.8 between eyes (NPi difference = 0.9).

The NPi scores for the left eye were not normally distributed (Shapiro–Wilk, w = 0.91, *p* = 0.03). Outliers were not removed due to clinical significance. Participants were placed into low, medium, or high NPi groups for each eye based on percentiles (Table 2). Table 3 includes median differences between low, medium and high NPi groups. Nonparametric Kruskal–Wallis H tests were used to determine differences between NPi scores for constriction percentage, maximum constriction velocity, minimum pupil size, and dilation velocity. The components of pupillometry were selected based on bivariate Pearson correlations. The constriction percentage of the right eye (r = 0.89, *p* < 0.001), minimum pupil size of the right eye (r = −0.61, *p* = 0.001), and maximum constriction velocity of the eye (r = 0.50, *p* < 0.01) were all significantly correlated with the NPi scores of the right eye. Similarly, the NPi scores of the left eye were associated with the constriction percentage of the left eye (r = 0.73, *p* < 0.001) and minimum pupil size of the left eye (r = −0.67, *p* < 0.001).

A Kruskal–Wallis H test demonstrated that the constriction percentage of the right eye had a significant effect on the NPi score for the right eye (*χ*^2^(2) = 17.84, *p* < 0.001, *E*^2^ = 0.69). The minimum pupil size of the right eye also affected the NPi score for the right eye (*χ*^2^(2) = 7.976, *p* = 0.019, *E*^2^ = 0.31). Constriction percentage R (η^2^ = 0.66) and minimum pupil size R (η^2^ = 0.25) explained 66% and 25% of the variance in the NPi score of the right eye, respectively. A post hoc Dunn test with Bonferroni adjustment showed significant differences in the constriction percentage of the right eye between low NPi and medium NPi groups (*p* < 0.01) and between low and high NPi groups (*p* < 0.001).

Similarly, in the left eye, the constriction percentage (*χ*^2^(2) = 10.315, *p* < 0.01, *E*^2^ = 0.40) and minimum pupil size (*χ*^2^(2) = 14.49, *p* < 0.01, *E*^2^ = 0.56) showed a significant difference in NPi. The constriction percentage of the left eye (η^2^ = 0.35) explained 35% of the variance in NPi, while the minimum pupil size of the left eye (η^2^ = 0.52) accounted for over 50% of the variance. Post hoc Dunn tests revealed significant differences between low NPi and medium (*p* = 0.45) and high NPi groups (*p* = 0.007) for the constriction percentage of the left eye. Table 4 shows mean pupillometry components in comparison to Npi scores for both left and right eyes.

A two-way random model assessed the intra-class correlation (ICC) for the VOMS score, convergence, and NPi. There was poor agreement between the measures (ICC = 0.22). Participants were split into two groups for normal or abnormal VOMS. Abnormal VOMS was defined as symptoms > 2. Kruskal–Wallis tests revealed no significant differences in the NPI for the right eye (*p* = 0.61) or left eye (*p* = 0.06) compared to VOMS. A component of the VOMS, the mean NPC, was calculated across three trials (1.86 ± 1.06 cm). Kruskal–Wallis H tests were used to determine if there were any differences in NPC for the NPi groups. No significant differences were found in NPC compared to NPi scores (*p* > 0.05).

One athlete sustained a concussion during the season and averaged an increased near point of convergence over three trials after concussion (0.75 vs. 1.42 cm) and an increase of ≥2 in VOMS symptoms when tested within 24 h after a concussion. The NPi did not change in the right eye and decreased by 0.1 in the left. As this athlete sustained a concussion at the end of the season, the athlete did not return to playing soccer.

## 4. Discussion

This study aimed to explore and compare quantitative pupillometry and VOMS in Division I female soccer athletes at baseline and to follow the athletes throughout the season. The quantitative pupillometry components of constriction percentage and minimum pupil size for both the left and right eyes were statistically significant, and abnormal findings were observed in collegiate soccer athletes at baseline, without any concussion symptoms. The results of the current study add to the evidence that subconcussive impacts in soccer disrupt oculomotor function in healthy athletes, even though the current study is limited in the number of participants.

When considering the usefulness of quantitative pupillometry, some background may be helpful for the reader. Pupillary reaction to light has been used for decades to indicate brain injury [27]. PEARL, or Pupils Equal And Reactive to Light, is a standard assessment used by physicians to evaluate a patient with suspected concussion in the emergency department or on the sidelines of an athletic event. Pupillometry measures allow for a standardized approach for evaluating PEARL by exposing the patient to light at a specific distance and brightness every time, as opposed to the use of a penlight by a healthcare worker in the emergency department or on the sidelines. Another distinct advantage is that pupillometry measurements on one athlete take less than 3 min. Pupillometry could save athletic healthcare providers valuable time with respect to baseline measurements and assessments of concussion.

In the current study, eight components were measured: NPi, maximum and minimum pupil size in millimeters, constriction percentage, latency, average constriction velocity (mm/s), maximum constriction velocity (mm/s), average dilation velocity (mm/s), and 75% recovery time (seconds). The NPi score is an index score based on the quantitative pupillometry components compared to normative values that range from 0 to 5. A clinical cutoff score of <3.0 or a difference between eyes of >0.7 for NPi has been suggested [28]. Researchers have suggested a cutoff of <0.8 mm/s for constriction velocity and <0.86 mm/s for dilation velocity [28]. Significant differences have been identified within the pupillometry components after injury, with constriction percentage, constriction velocity, and dilation velocity showing the most consistent differences when comparing controls to concussed individuals [29]. Military personnel with blast-induced or other forms of mild traumatic brain injury have shown decreased constriction and dilation velocity compared to healthy age-matched controls in a small sample [20]. More research is needed regarding quantitative pupillometry in the collegiate-aged athletic population, which triggered interest in the current study.

In the current study, quantitative pupillometry components were abnormal even though none of the athletes had a concussion. Oddo et al. noted that an NPi score of 3 and below for patients admitted to an intensive care unit after severe brain injury was compared to Glasgow Coma Scale scores to determine poor neurological outcomes. The soccer athletes in the current study had variable NPi scores, with nine athletes having <3.8 NPi and two athletes having NPi scores of 3 without any symptoms or complaints of a concussion at baseline testing. As the NPi is a composite score of eight separate components, perhaps it is more important to investigate the components, and the NPi score needs to be discarded as clinically irrelevant. Recently, Dengler et al. [30] investigated 1300 cadets at baseline compared to 68 cadets who sustained a concussion. Dengler et al. [30] reported the significant components of constriction diameter, dilation velocity, and pupillary constriction. Dengler et al. [30] suggest that quantitative pupillometry has the potential to assist in predicting symptom severity and duration before an individual’s return to activity. Future studies should consider providing cutoff scores for components specific to athletes and the non-athletic population, such as older adults.

Subconcussive impacts have recently been investigated to determine the effects on the brain in soccer heading [31,32,33,34]. Grijalva et al. [31] examined 20 collegiate soccer athletes and measured blood flow velocity through transcranial ultrasound and oxyhemoglobin concentrations using portable functional near-infrared spectroscopy imaging to identify pre- and post-header measurements minutes after the headers. Grijalva et al. [31] also measured balance and dual-task neurocognitive testing pre- and post-soccer headers. They reported variations in brain oxygenation, significant balance impairment, and decreased neurocognitive function scores immediately after 10 soccer headers [31]. Kawata et al. [32] examined the effect of 10 soccer headers on near-point convergence scores in 20 healthy soccer players. Kawata et al. [32] assessed near-point convergence before the headers, less than 1 h after the headers, and 24 h after the headers. Kawata et al. [32] reported that 10 controlled soccer headers projected at a speed of 11.2 m/s significantly impacted near point of convergence scores less than 1 h after the headers and 24 h after the headers [32]. Kawata et al. did not investigate or report symptoms in this study. Perhaps this is because the soccer players in the Kawata et al. study did not have any symptoms and could not recognize the effect of soccer headers, yet the VOMS near point of convergence test is sensitive enough to identify differences. Future studies could consider the pre- and post-soccer header impact on pupillometry components. The current study only measured baseline pupillometry but included all VOMS components and not just NPC, as a comprehensive concussion assessment should include objective and subjective measures.

The VOMS scores were not significantly different from pupillometry components. VOMS and pupillometry measure different visual domains, but research suggests that both of these oculomotor tests may be disrupted in a concussion. The current study compared the two measures to see if there were associations. Only two soccer athletes had abnormal VOMS scores. The lack of differences suggests that pupillometry measures are separate from the VOMS. As none of the soccer players reported a concussion during baseline testing, this reinforces the use of VOMS for baseline testing. Future studies should focus on the pupillometry components and consider not using the NPi. One soccer athlete who had a diagnosed concussion presented with abnormal VOMS scores 24 h after her concussion, with complaints during the visual motion sensitivity, vertical and horizontal saccade, and convergence tests. This athlete had a normal NPi score but presented abnormal bilateral constriction percentages and abnormal minimum bilateral pupil size measures. As this was the end of the soccer season, there was no follow-up regarding testing for this one concussed soccer athlete, as she was not returning to play or practice.

The current study has some limitations. As this study only followed soccer athletes, the number of participants and the number of concussions were limited, and we did not include a control group of non-athletes. Future studies could consider comparisons of soccer athletes in a long-term study evaluating preseason, midseason, and postseason testing to compare differences and effects across a season. A comparison of the NPi and VOMS scores of athletes across different sports and adolescents would be ideal. Another limitation is that it is unknown to the researcher if any of the athletes were taking medications that may have influenced pupillometry measures, such as attention deficit medications, before baseline testing. This should be asked in future studies to determine the medication impact on the pupil’s parasympathetic control via pupillometry. Future studies should include a control group to compare VOMS and pupillometry components in age-matched females.

## 5. Conclusions

Research on pupillometry is limited to the collegiate athlete population, but it seems worth investigating as it is an efficient test that is easy to administer in a large group of athletes. Ideally, cutoff scores of the most essential components of pupillometry should be determined for specific populations. The results of the current study add to the evidence that soccer disrupts oculomotor function in a small sample of healthy female athletes, even before signs of a concussion, and impacts symptoms during VOMS testing.

## Figures and Tables

**Table 1:** Means and standard deviations for quantitative pupillometry and Vestibular Ocular Motor Screening (VOMS) at baseline (n = 26).

	Mean and Standard Deviation	Right Eye	Left Eye
NPi		3.85 ± 0.39	3.97 ± 0.42
Pupil Size (mm)		5.41 ± 0.79	5.26 ± 0.79
Minimum Pupil Size (mm)		3.65 ± 0.59	3.47 ± 0.53
Constriction Percentage (%)		32.63 ± 4.33	33.70 ± 5.24
Constriction Velocity (mm/s)		2.65 ± 0.50	2.90 ± 0.54
Maximum Constriction Velocity (mm/s)		4.21 ± 0.68	4.50 ± 0.87
Latency (s)		0.22 ± 0.02	0.21 ± 0.02
Dilation Velocity (mm/s)		1.12 ± 0.24	1.18 ± 0.24
NPC Trial 1	1.89 ± 1.00		
NPC Trial 2	1.84 ± 1.02		
NPC Trial 3	1.84 ± 1.25		
NPC Trial Average	1.86 ± 1.06		
VOMS Total Score	1.04 ± 3.48		

**Table 2:** Neurological Pupillary Index (NPi) score cutoffs for high, medium, and low groups (n = 26).

	Right Eye	Number	Left Eye	Number
Low NPi	<3.8	n = 10	<3.87	n = 9
Medium NPi	3.8–4.1	n = 9	3.87–4.1	n = 8
High NPi	>4.1	n = 8	>4.1	n = 9

The cutoff scores are based on each group’s 33.33 and 66.67 percentiles.

**Table 3:** Median differences between Neurological Pupillary Index (NPI) scores (n = 26).

	Low NPi	Medium NPi	High NPi	Significance
Constriction %, Right	28.50	34.00	36.50	*p* < 0.001 *
Max Constriction Velocity, Right	3.70	4.67	4.54	*p* = 0.05
Min Pupil Size, Right	3.86	3.75	3.14	*p* = 0.02 *
Dilation Velocity, Right	1.09	1.16	1.13	*p* = 0.88
Constriction %, Left	30.00	37.00	37.50	*p* = 0.006 *
Max Constriction Velocity	4.27	5.06	4.41	*p* = 0.29
Min Pupil Size, Left	3.87	3.77	2.91	*p* = 0.001 *
Dilation Velocity, Left	1.12	1.39	1.13	*p* = 0.311

* Statistical significance *p* < 0.05.

**Table 4:** Paired *t*-test of mean pupillometry component scores and Neurological Pupillary Index (NPI) scores (n = 26).

Pupillometry Components	Mean and Standard Deviation
Right NPI	3.84 (0.38)
Right Pupil Size	5.42 (0.79)
Right NPI	3.84 (0.38)
Right Minimum Pupil Size at Constriction	3.66 (0.59)
Right NPI	3.84 (0.38)
Right Constriction Percentage Change	0.325 (0.04)
Right NPI	3.84 (0.38)
Right Constriction Velocity	2.63 (0.49)
Right NPI	3.84 (0.38)
Right Maximum Constriction Velocity	4.18 (0.67)
Right NPI	3.84 (0.38)
Right Constriction Latency	0.220 (0.02)
Right NPI	3.84 (0.38)
Right Dilation Velocity	1.10 (0.25)
Left NPI	3.95 (0.43)
Left Pupil Size	5.27 (0.81)
Left NPI	3.95 (0.43)
Left Minimum Pupil Size at Constriction	3.49 (0.53)
Left NPI	3.95 (0.43)
Left Constriction Percentage Change	0.35 (0.05)
Left NPI	3.95 (0.43)
Left Constriction Velocity	2.90 (0.55)
Left NPI	3.95 (0.43)
Left Maximum Constriction Velocity	4.50 (0.89)
Left NPI	3.95 (0.43)
Left Constriction Latency	0.21 (0.02)
Left NPI	3.95 (0.43)
Left Dilation Velocity	1.17 (0.24)

## Data Availability

Data are available upon reasonable request to the corresponding author.

## Abbreviations

The following abbreviations are used in this manuscript:

VOMS	Vestibular Ocular Motor Screening Test;
NPI	Neurological Pupillary Index.
